# SARS Epidemiology Modeling

**DOI:** 10.3201/eid1006.031023

**Published:** 2004-06

**Authors:** Ying-Hen Hsieh, Jen-Yu Lee, Hsiao-Ling Chang

**Affiliations:** * National Chung Hsing University, Taichung, Taiwan;; † Center for Disease Control, Taipei, Taiwan

**Keywords:** SARS, epidemiology, mathematical model, Taiwan, logistic-type model

**To the Editor:** To assess the effectiveness of intervention measures during the recent severe acute respiratory syndrome (SARS) pandemic, Zhou and Yan (*1*) used Richards model, a logistic-type model, to fit the cumulative number of SARS cases reported daily in Singapore, Hong Kong, and Beijing. The key to using mathematical models for SARS epidemiology is understanding the models (*2*). In the Richards model (*1*), the function *F(S)* in the model was described as measuring "the effectiveness of intervention measures." The parameters in *F(S)*, namely the maximum cases load *K* and the exponent of deviation *a*, depict the actual progression of the epidemic as described by the reported data. In other words, the parameter estimates are used to quantify end results of the intervention measures implemented during the outbreaks. Simply put, the all-important question of "what if?" was not answered by their result. To gauge the effectiveness of intervention measures, one should consider a more complicated model with variable maximum case load and growth rate (*r*) that highlights the time-varying nature of an epidemic and its dependence on the intervention measures implemented during the epidemic.

Predicting the trend of an epidemic from limited data during early stages of the epidemic is often futile and sometimes misleading (*3*). Nevertheless, early prediction of the magnitude of an epidemic outbreak is immeasurably more important than retrospective studies. But how early is too early? Intuitively, the cumulative case curve will always be S-shaped and well-described by a logistic-type model. The essential factor is the time when the inflection of the cumulative case curve occurs, i.e., the moment when a rapid increase in case numbers is replaced by a slower increase. Since the inflection point, approximated by *t_m_* (*1*), dictates the point in time when the rate of increase of cumulative case numbers reaches its maximum, the moment marks the key turning point when the spread of the disease starts to decline. As long as the data include this inflection point and a time interval shortly after, the curve fitting and predicting future case number will be reasonably accurate.

To illustrate this point more precisely, the cumulative SARS case data by onset date in Taiwan were obtained from the SARS databank of Taiwan Center for Disease Control. The data cover the time from February 25, 2003, the onset date of the first confirmed SARS case, to June 15, 2003, the onset date of the last confirmed case; a total of 346 SARS cases were confirmed during the 2003 outbreak in Taiwan (*4*). The cumulative case data are fitted to the cumulative case function *S(t)* in Richards model with the initial time *t_0_* = 0 being February 25 and the initial case number *S_0_* = *S(0)* = 1. Description of the model, as well as the result of the parameter estimation, is shown in Tables A1-A6. The estimates for the parameters are *r* = 0.136 (95% confidence interval [CI] 0.121 to 0.150), *K* = 343.4 (95% CI 339.7 to 347.1), *a* = 1.07 (95% CI 0.80 to1.35), and the approximate inflection point at t_m_ = 66.62 (95% CI 63.9 to 69.3) with adjusted r^2^ >0.998, p < 0.0001 for the goodness-of-fit of the model (Figure). The result indicates that the inflection point occurred on May 3, and the estimate for the maximum case number *K* = 343.3 is 0.8% off the actual total case numbers.

**Figure:**
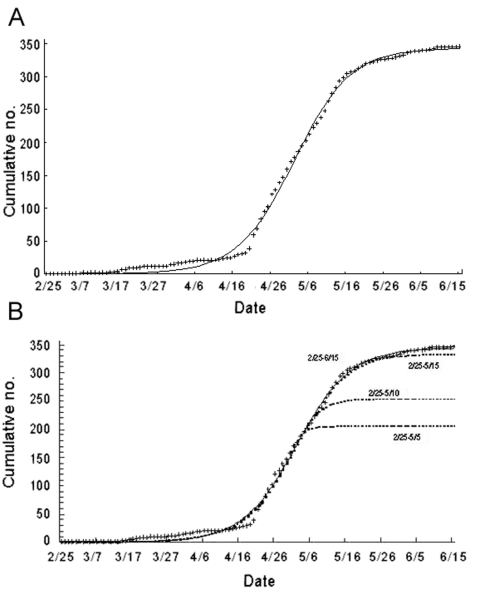
SARS cases, Taiwan, 2003, using Richards model; t = real data. A, confirmed cases; B, estimated cases using the truncated data.

Moreover, the case number data are sorted by onset date. Given a mean SARS incubation of 5 days (4–6 days) (*5*), the inflection point for SARS in Taiwan could be traced back to 5 days before May 3, namely April 28. On April 26, the first SARS patient in Taiwan died. Starting April 28, the government implemented a series of strict intervention measures, including household quarantine of all travelers from affected areas (*6*). In retrospect, April 28 was indeed the turning point of the SARS outbreak in Taiwan.

To address making projections during an ongoing epidemic, we used the same dataset but used various time intervals (all starting February 25) but truncated at various dates around the inflection point of May 3. The resulting parameter estimates are given in Tables A1-A6. For the truncated data ending on April 28 before the inflection, an unreasonable estimate of *K* = 875.8 was obtained. However, if we use the data ending on May 5, May 10, May 15, and May 20, we obtain estimates of *K* = 204.9, 253.1, 334.2, and 342.1, respectively. These estimates improve as we move further past the inflection time of May 3 (Figure). Moreover, the last estimate, using data from February 25–May 20 only, produces a 1.1% error from the eventual cumulative case number of 346, with 95% CI of 321.5 to 362.6. This retrospective exercise demonstrates that if the cumulative case data used for predictive purpose during an outbreak contain information on the inflection point and approximately 2 weeks afterwards, the estimate for the total case number can be obtained with accuracy, well before the date of the last reported case. This procedure may be immensely useful for deciding future public health policies although correctly determining the true inflection point during a real ongoing epidemic calls for scrutiny and judicious use of the model, as with all mathematical epidemic models.

## Appendix

**Table A1:** Study 1, patient characteristics, methicillin-resistant *Staphylococcus aureus* (MRSA), controls not infected with *S. aureus* and controls with methicillin-susceptible *S. aureus* (MSSA) surgical site infections, bivariable analyses

Variable	Cases, MRSA (%) (n = 121)	Controls, uninfected patients (%) (n = 193)	p value, (MRSA vs. uninfected controls)	Controls, MSSA (%) (n = 165)	p value (MRSA vs. MSSA)
Age, mean ± SD, y	63.9 ± 15.4	57.3 ± 18.3	0.001	55.1 ± 17.4	<0.001
Male sex	55 (45.5)	92 (42.7)	0.73	90 (54.6)	0.15
Coexisting conditions					
Diabetes mellitus	59 (48.8)	66 (34.2)	0.01	57 (34.6)	0.02
Hematologic disorder	1 (0.8)	1 (0.5)	1.00	2 (1.2)	1.00
HIV infection	0 (0.0)	1 (0.5)	1.00	0	1.00
Hypertension	64 (52.9)	75 (38.9)	0.02	80 (48.5)	0.48
Liver disease	4 (3.3)	1 (0.5)	0.07	2 (1.2)	0.25
Malignancy	15 (12.4)	14 (7.3)	0.16	13 (7.9)	0.23
Obesity	10 (8.3)	12 (6.2)	0.50	18 (10.9)	0.55
Peripheral vascular disease	12 (9.9)	3 (1.6)	0.002	9 (5.5)	0.17
Pulmonary disease	21 (17.4)	23 (11.9)	0.19	32 (19.4)	0.76
Renal disease	19 (15.7)	9 (4.7)	0.002	13 (7.9)	0.06
Transplant	1 (0.8)	0	0.39	0	0.42
Tobacco use	16 (13.2)	20 (10.4)	0.47	24 (14.6)	0.86
Alcohol abuse	4 (3.3)	2 (1.0)	0.21	6 (3.6)	1.00
Hospital-related risk factors					
Treatment at the academic tertiary care hospital	94 (77.8)	125 (64.8)	0.02	109 (66.1)	0.04
LOS before surgery, median, IQR	1, 0–4	0, 0–3	0.02	0, 0–2	0.01
LOS before culture, median, IQR	8, 5–14	NA	NA	5, 3–10	<0.001
Proportion of patients with an ICU stay before surgery	11 (9.1)	13 (7.9)	0.83	18 (9.3)	1.0
ASA score, median, IQR	3, 3–4	3, 2–4	0.03	3, 2–4	0.15
Duration of surgery (min), median, IQR	240, 166–305	194, 113–276	0.004	202, 116–285	0.01
Wound class, median, IQR	1, 1–1	1, 1–1	0.82	1, 1–1	0.36
NNIS Risk Index, median, IQR	1, 1–2	1, 1–1	0.002	1, 1–2	0.06

^a^LOS, length of stay; IQR, interquartile range; ASA, American Society of Anesthesiologists-Physical Status score; NNIS, National Nosocomial Infections Surveillance System.

**Table A2:** Study 1: Adjusted outcomes models for methicillin-resistant *Staphylococcus aureus* (MRSA) surgical site infection (SSI) compared to uninfected control patients^a^

Variable	Deaths	Length of stay^b^	Cost^c^
	OR (95% CI)	OR^d^ (95% CI)	OR (95% CI)
MRSA	11.4 (2.8 to 34.9)	3.2 (2.7 to 3.7)	2.2 (2.0 to 2.6)
ASA score^e,f^		1.3 (1.2 to 1.5)	ASA score = 4 3.7 (1.5 to 8.9)
			ASA score = 2 2.0 (1.4 to 2.9)
			ASA score = 3 3.0 (2.1 to 4.3)
			ASA Score = 4 4.1 (2.8 to 6.0)
>73 y of age	4.8 (2.0 to 11.6)		
Operative duration (min)^g^			
211–400		(0.9 to 1.3)	1.4 (1.2 to 1.7)
401–590		1.7 (1.2 to 2.4)	2.2 (1.6 to 3.1)
>590		1.8 (1.1 to 2.9)	2.6 (1.6 to 4.0)
Length of stay before surgery^h^			
7–13 d		1.6 (1.1 to 2.1)	1.7 (1.3 to 2.3)
14–20 d		3.6 (1.4 to 9.6)	5.6 (2.3 to 13.4)
>20 d		0.7 (0.2 to 2.6)	1.2 (0.3 to 4.3)
Intensive care unit stay before surgery			1.5 (1.2 to 2.0)
Tertiary care hospital			1.5 (1.2 to 1.7)

^a^OR, odds ratio; CI, confidence interval; ASA, American Society of Anesthesiologists -Physical Status. ^b^Model includes the following confounding variables: admission to the tertiary care hospital, diabetes, and renal disease. ^c^Model includes the following confounding variable: renal disease. ^d^For length of hospital stay and cost, OR represents multiplicative effect ^e^Length of stay increases by 1.3-fold for each point increase in ASA score. ^f^For cost, reference category is ASA score = 1. ^g^Reference category is operative duration < 211 min. ^h^Reference category is length of stay before surgery < 7 d.

**Table A3:** Study 1, adjusted outcomes models for methicillin-resistant *Staphylococcus. aureus* (MRSA) surgical site infections (SSI) compared to patients with methicillin-resistant *S. aureus* (MSSA) SSI^a^

	Deaths^b^	Length of Stay^c^	Cost^d^
Variable	OR (95% CI)	OR (95% CI)^e^	OR^e^ (95% CI)
MRSA	3.4 (1.5 to 7.7)	1.2 (1.0 to 1.5)	1.2 (1.0 to 1.4)
ASA score^f^	ASA score = 4 5.1 (2.1 to12.5)	ASA score = 2 0.9 (0.5 to 1.7)	ASA score = 2 1.0 (0.7 to 1.5)
		ASA score = 3 1.6 (0.9 to 2.9)	ASA score = 3 1.4 (1.0 to 2.1)
		Asa score = 4 1.8 (1.0 to 3.5)	ASA score = 4 2.1 (1.4 to 3.2)
Age > 61 years	3.0 (1.2 to 7.3)		
Operative duration, min^g^			
206–381		1.3 (1.0 to 1.6)	1.4 (1.1 to 1.6)
382–557		1.3 (0.8 to 2.1)	1.8 (1.3 to 2.5)
>557		1.1 (0.5 to 2.6)	1.6 (0.9 to 2.8)
Length (d) of stay before infection^h^			
11–20		1.4 (1.0 to 1.8)	1.6 (1.3 to 2.0)
21–30		1.6 (1.0 to 2.7)	1.7 (1.2 to 2.5)
>30		1.3 (0.5 to 3.1)	1.8 (0.9 to 3.8)
Renal disease		1.5 (1.0 to 2.2)	
Length (d) of intensive care unit stay before infection^i^			
8–14			1.8 (1.1, 2.8)
15–21			2.1 (1.1, 8.8)
>21			1.9 (0.4, 8.0)
Tertiary care hospital			1.3 (1.1, 1.6)

^a^OR, odds ratio; CI, confidence interval; ASA, American Society of Anesthesiologists -Physical Status. ^b^Model includes the following confounding variable: operative duration >222 min. ^c^Model includes the following confounding variables: admission to tertiary care hospital and diabetes. ^d^Model includes the following confounding variables: diabetes and renal disease. ^e^For length of hospital stay and cost, OR represents multiplicative effect. ^f^For deaths, reference category is ASA score < 1; for length of stay and cost, reference category is ASA score = 1. ^g^Reference category is operative duration < 206 min. ^h^Reference category is length of stay prior to infection < 11 d. ^i^Reference category is intensive care unit length of stay prior to infection < 8 d.

**Table A4:** Study 2, patient characteristics, vancomycin-resistant enterococci (VRE) wound infections, controls not infected with enterococci, and controls with vancomycin-susceptible enterococci (VSE) wound infections, bivariate analyses

Variable	Cases, VRE wound (%) (n = 99)	Controls, not infected (%) (n = 280)	P Value (VRE vs. controls not infected)	Controls, VSE (%) (n = 213)	p value (VRE vs. VSE)
Age, mean (y)	60.3	63.6	0.09	59.1	0.51
Sex (female)	46 (46)	124 (44.3)	0.7	127 (59.6)	0.03
Main diagnosis					
Orthopedic condition	11 (11)	30 (10.7)		18 (8.4)	
Cardiovascular condition	25 (25)	117 (41)		61 (28.6)	
Endocrine disorder	3 (3)	6 (2.1)		4 (1.9)	
Gastrointestinal disorder	25 (25)	60 (21.4)		62 (29.1)	
Genitourinary disorder	6 (6)	12 (4.2)		9 (4.3)	
Infectious disease	16 (16)	6 (2.1)		20 (9.4)	
Hematologic disease	0 (0)	2 (.7)		0	
Neurologic disease	11 (11)	32 (11.4)		34 (16)	
Pulmonary disease	2 (2)	14 (5)		5 (2.4)	
Coexisting conditions					
Cardiovascular disease	73 (74)	204 (72.9)	0.86	150 (70.4)	0.55
Lung disease	11 (11)	33 (11.7)	0.9	26 (12.2)	0.78
Diabetes mellitus	67 (67.7)	139 (49.6)	0.002	127 (59.6)	0.17
Organ transplant recipient	14 (14)	21 (7.5)	0.08	18 (8.4)	0.12
Renal disease	18 (18.2)	39 (14)	0.7	28 (13.2)	0.24
Malignancy	7 (7.1)	27 (9.6)	0.5	32 (15)	0.05
AIDS	2 (2)	2 (0.7)	0.27	0	0.1
Hepatobiliary disease	16 (16.6)	40 (14.3)	0.8	31 (14.5)	0.71
Charlson comorbidity score, mean	3.17	2.66	0.07		
Hospital-related risk factors					
Transfer from another institution	34 (34.3)	102 (36.4)	0.5	34 (16)	<0.001
Surgery	29 (29.3)	94 (33.6)	0.08	90 (42.3)	0.03
Admission to ICU	26 (26.2)	58 (20.7)	0.9	53 (33.3)	0.8

**Table A5:** Study 2, adjusted outcomes models for vancomycin-resistant enterococcus (VRE) wound infection compared to uninfected control patients^a^

Variable	Deaths^b^	Variable	Length of Stay^c^	Variable	Cost^d^
	OR (95% CI)		OR^e^ (95% CI)		OR^e^ (95% CI)
VRE infection	2.0 (0.8 to 5.2)	VRE infection	1.8 (1.3 to 2.4)	VRE infection	1.5 (1.3, 1.8)
		Transfer from another hospital	1.5 (1.2 to 1.9)	Surgery^e^	1.4 (1.1, 1.8)
		Renal disease	2.0 (1.5 to 2.7)		
		Malignancy	0.7 (0.5 to 0.9)		
		Intensive care unit stay^f^	2.3 (1.6 to 3.3)		

^a^OR, odds ratio; CI, confidence interval. ^b^Model includes the following confounding variables: intensive care unit (ICU) stay and number of coexisting conditions. ^c^Model includes the following confounding variable: propensity score (i.e., likelihood of being a VRE case). ^d^Model includes the following confounding variables: propensity score [i.e., likelihood of being a VRE case (Appendix)] and length of stay before infection (index date for controls). ^e^For length of hospital stay and cost, OR represents multiplicative effect. ^f^Before infection for cases and before index date for controls.

**Table A6:** Study 2, adjusted outcomes models for vancomycin-resistant enterococcus (VRE) wound infection compared to control patients with wound infection due to vancomycin-susceptible enterococcus (VSE)^a^

Variable	Deaths^b^		Length of Stay^c^		Cost^d^
	Odds Ratio (OR) (95% Confidence Interval [CI])	Variable	OR^e^ (95% CI)	Variable	OR^e^ (95% CI)
VRE	2.5 (1.1, 6.1)	VRE	1.1 (0.9, 1.4)	VRE	1.4 (1.2, 1.6)
Intensive care unit stay (ICU)^f^	9.0 (3.0, 27.4)	ICU stay^f^	1.8 (1.3, 2.5)	Surgery^f^	1.2 (1.1, 1.3)

^a^OR, odds ratio; CI, confidence interval; ICU, intensive care unit. ^b^Model includes the following confounding variables: gender and surgery before infection. ^c^Model includes the following confounding variable: malignancy and length of stay before infection. ^d^Model includes the following confounding variables: length of stay before cohort inclusion. ^e^For length of hospital stay and cost, OR represents multiplicative effect. ^f^Before infection for cases and before index date for controls.

## References

[1] Zhou G, Yan G. Severe acute respiratory syndrome epidemic in Asia. Emerg Infect Dis. 2003;9:1608–10. 14720403 10.3201/eid0912.030382 PMC3034341

[2] Hsieh YH, Chen CWS. Re: Mathematical modeling of SARS: cautious in all our movements. J Epidem Com Health [serial on the Internet]. 2003 [cited 2003 Nov 18]. Available from: http://jech.bmjjournals.com/cgi/eletters/57/6/DC1#66

[3] Razum O, Becher H, Kapaun A, Junghanss T. SARS, lay epidemiology, and fear. Lancet. 2003;361:1739–40. 10.1016/S0140-6736(03)13335-1 12767754 PMC7124702

[4] World Health Organization. Summary of probable SARS cases with onset of illness from 1 November 2002 to 31 July 2003 [monograph on the Internet]. [cited 2003 Sep 26]. Available from: http://www.who.int/csr/sars/country/table2003_09_23/en/

[5] World Health Organization. Consensus document on the epidemiology of severe acute respiratory syndrome (SARS) [monograph on the Internet]. [cited 2003 Oct 17]. Available from: http://www.who.int/csr/sars/en/WHOconsensus.pdf

[6] Lee ML, Chen CJ, Su IJ, Chen KT, Yeh CC, King CC, Use of quarantine to prevent transmission of severe acute respiratory syndrome—Taiwan, 2003. MMWR Morb Mortal Wkly Rep. 2003;52:680–3. 12881699

