# Supplementary material for: SARS Epidemiology Modeling
Source: Emerg Infect Dis. 2004 Jun;10(6):1165–7. doi: 10.3201/eid1006.031023 (PMC3323156; doi:10.3201/eid1006.031023)
Supplement: Related texts

# Reply: SARS Epidemiology Modeling

**DOI:** 10.3201/eid1006.040173

**Authors:** Guofa Zhou, Guiyan Yan

**Affiliations:** * State University of New York, Buffalo, New York, USA

**In Reply:** Our analysis of the dynamics of reported severe acute respiratory syndrome (SARS) clinical cases was conducted in May 2003 during the height of the public panic (*1*). Our primary goal in that study was to predict "when the epidemic might be brought under control if the current intervention measures were continued" (*1*). We used the Richards model and successfully predicted the epidemic cessation dates in Beijing, Hong Kong, and Singapore. Our predicted total number of SARS cases was close to the actual number of cases. In addition, we estimated the basic reproductive rate (R_0_) of SARS infection, and our estimates based on the deterministic model were similar to those based on stochastic models (*2**,**3*). Therefore, our analysis provided useful information on the epidemiologic characteristic of SARS infections in three major Asian cities.

Hsieh et al. (*4*) commented that our article did not address the effect that specific intervention measures might have on the dynamics of SARS infection. Our study was not intended to measure this. As we stated in our article, "the transmission mechanism of the coronavirus that causes SARS and the epidemiologic determinants of spread of the virus are poorly understood." Any models built on these unknowns are not suitable for assessing the effects of specific intervention measures. A method suggested by Hsieh et al. (*4*) to merely "consider a more complicated model with variable maximum case load and growth rate" will not answer the question to any extent.

The retrospective analysis of SARS case dynamics in Taiwan by Hsieh et al. (*4*) found that "as long as the data include this inflection point and time interval shortly after, the curve fitting and predicting future case number will be reasonably accurate." This notion holds only if the true inflection point is known before an epidemic ends. The main difficulty is how the true inflection point is correctly determined, as noted by Hsieh et al. (*4*). The time when inflection occurs varies tremendously if truncated data of cumulative SARS case numbers are used. To illustrate this point, we used the cumulative number of reported probable SARS cases in Hong Kong, starting March 17, 2003, but truncated at various dates, and calculated the date when inflection occurred (Table). For example, if the data period from the onset date (March 17, 2003) to the last case reported (June 12, 2003) was used, the date when inflection would occur was estimated as March 19, 2003. If the truncated data ending April 9, April 16, April 30, May 14, and May 28, 2003, were used, the dates when inflection would occur were estimated as April 2, February 7, March 3, March 23, and April 2, 2003, respectively (Table). Clearly, inflection point dates became a moving target as the epidemic progressed. When truncated data ending April 9, April 16, April 30, May 14, and May 28, 2003, were used, the corresponding estimated maximum numbers of cumulative cases (*K*) were 1,107, 1,907, 1,819, 1,749, and 1,733, respectively. Estimation of *K* improved when the data period used for prediction was at least one month past the March 19 inflection point obtained from the entire epidemic period. This analysis highlights the difficulty in identifying an optimal inflection point for prediction purposes during an ongoing epidemic when only a partial cumulative case number is available.

**Table:** Predicted inflection point and dates when inflection occurs based on truncated data of cumulative number of reported severe acute respiratory syndrome cases in Hong Kong

Data period (ending date)	*t_m_* ^a^	Date^b^	*K* ^c^	*r* ^d^	α^e^
April 9, 2003	16.62	April 2, 2003	1,107	0.20	0.74
April 16, 2003	–40.79	February 7, 2003	1,907	0.07	52.11
April 30, 2003	–13.52	March 3, 2003	1,819	0.07	10.21
May 14, 2003	6.80	March 23, 2003	1,749	0.09	2.84
May 28, 2003	17.31	April 2, 2003	1,733	0.10	1.38
June 12, 2003	2.63	March 19, 2003	1,751	0.09	3.77

^a^*t_m_* is the inflection point of the model. ^b^Date refers to the date when inflection occurs. ^c^*K* is the predicted maximum number of cumulative cases. ^d^*r* is the intrinsic growth rate. ^e^α measures the extent of deviation of S-shaped dynamics from the classic logistic growth curve.

We fully agree with Hsieh et al. (*4*) that the quantitative assessment of the effectiveness of public health intervention measures for SARS is a difficult task for modelers. To make models useful for assessing the effects of specific intervention measures and for predicting the future dynamics during an ongoing epidemic, we need improved knowledge on the transmission mechanisms, pathogenesis, and the epidemiologic determinants of the spread of the virus. Any retrospective analysis of the 2003 SARS epidemic that improves our knowledge of SARS epidemiology is welcome.

## References

[1] Zhou G, Yan G. Severe acute respiratory syndrome epidemic in Asia. Emerg Infect Dis. 2003;9:1608–10.1472040310.3201/eid0912.030382PMC3034341

[2] Lipsitch M, Cohen T, Cooper B, Robins JM, Ma S, James L, Transmission dynamics and control of severe acute respiratory syndrome. Science. 2003;300:1966–70. 10.1126/science.108661612766207PMC2760158

[3] Riley S, Fraser C, Donnelly CA, Ghani AC, Abu-Raddad LJ, Hedley AJ, Transmission dynamics of the etiological agent of SARS in Hong Kong: impact of public health interventions. Science. 2003;300:1961–6. 10.1126/science.108647812766206

[4] Hsieh YH, Lee JY, Chang HL. SARS epidemiology and cumulative case curve. Emerg Infect Dis. 2004;10:1165–7. 10.3201/eid1006.03102315224675PMC3323156
